# Fatigue as Mediator Factor in PTSD-Symptoms after Allogeneic Hematopoietic Stem Cell Transplantation

**DOI:** 10.3390/jcm12082756

**Published:** 2023-04-07

**Authors:** Giulia Di Francesco, Filippo Cieri, Roberto Esposito, Pierpaola Sciarra, Valeria Ballarini, Mauro Di Ianni, Stella Santarone

**Affiliations:** 1 Hematology Unit, Department of Oncology-Hematology, Pescara Hospital, 65100 Pescara, Italy; 2 Department of Neurology, Cleveland Clinic Lou Ruvo Center for Brain Health, Las Vegas, NV 89106, USA; 3 Clinica Diagnostica Titano (Bianalisi), 47891 Falciano, San Marino; 4 Azienda Sanitaria Territoriale (AST1), 61121 Pesaro, Italy; 5 Bone Marrow Transplant Unit, Department of Oncology-Hematology, Pescara Hospital, 65100 Pescara, Italy

**Keywords:** post-traumatic stress disorder, quality of life, fatigue, allogeneic haemopoietic stem cell transplantation, hematological malignancies

## Abstract

Background: Allogeneic Hematopoietic Stem Cell Transplantation (HSCT) is a valid treatment for hematological oncological or metabolic diseases. Despite its therapeutic efficacy, it is an aggressive treatment that impacts negatively on quality of life (QoL) and may result in Post-Traumatic Stress Disorder (PTSD) symptoms. The aim of this study is to explore rates and risk factors for PTSD symptoms, and fatigue in post-HSCT patients with hematological malignancies. Methods: A total of 123 patients after HSCT were evaluated for PTSD symptoms, QoL and fatigue. PTSD symptoms were assessed with the Impact of Event Scale- Revised (IES-R), QoL was measured with Functional Assessment of Cancer Therapy-Bone Marrow Transplant (FACT-BMT) and fatigue symptoms were assessed with Functional Assessment of Chronic Illness Therapy-Fatigue (FACIT-F). Results: A total of 58.54% of the sample developed PTSD symptoms after transplant. Patients with PTSD symptoms reported significantly lower QoL total scores and significantly higher fatigue than those without PTSD symptoms (*p* < 0.001). The SEM analysis showed that worse QoL and fatigue affected PTSD symptomatology along different pathways. Fatigue was found as a major influencing factor of PTSD symptoms directly (β = 0.31 **), while QoL only through the mediation of fatigue at a lesser extent. (β = 0.33 *). Conclusions: Our findings indicate that QoL is a concurrent causative factor to the development of PTSD symptomatology through the mediating role of fatigue. Innovative interventions before transplantation to prevent PTSD symptoms should be investigated to improve survival and QoL in patients.

## 1. Introduction

The Allogeneic Hematopoietic Stem Cell Transplantation (HSCT) represents a valid treatment for patients suffering from hematological oncological or metabolic diseases. Despite its therapeutic efficacy, it is an aggressive treatment, with high risk of morbidity and mortality. Among the adverse outcomes, we can find graft versus-host-disease or immunosuppressive medication with a strong negative impact on QoL. Despite transplantation rapidly improving, toxicity and invasiveness still leads to a relatively high mortality rate [1]. Medical conditions related to HSCT may result in substantial psychological distress. In fact, this treatment is considered a traumatic event [2,3]. The DSM-5 (American Psychiatric Association, Washington, DC, USA, 2013) criteria for PTSD include, first, direct, or indirect exposure to a traumatic event, followed by symptoms in four categories: intrusion, avoidance, negative changes in thoughts and mood, and changes in arousal and reactivity [4].

### 1.1. PTSD

Kuba and colleagues found that conditions such as uncertainty, appearance and sexuality, and health burden were correlated to PTSD symptoms in 57% of patients after HSCT [5]. These patients can relive cancer and treatment experience with nightmares, flashbacks, or continuous thoughts about the treatment period. The trauma sequelae occur over an extended period following the medical procedure but also as a result of the anticipation of undergoing to HSCT [2]. The period of trauma appraisal (perceived life threat) may therefore be prolonged, thus delaying psychological recovery. A decline in QoL, with an increase in depressive symptoms during the HSCT hospitalization, are key predictors of PTSD symptoms [6]. 

Although the number of transplantations has been increasing over the past few years, with a post-transplant survival exceeding 85–90% in the first year, and 70–75% at 5 years [7,8], general psychophysical health conditions of this specific population are underrepresented and underestimated.

### 1.2. Quality of Life 

These patients have a significant risk of acute complications and late side effects with a negative impact on patients’ Quality of Life (QoL) [9,10], and a greater likelihood of development of a clinical diagnosis of PTSD, or sub-threshold PTSD symptoms [11,12]. QoL has been described as a dynamic and multifaceted concept related to several dimensions of well-being, including physical, emotional, functional, and social wellness [13]. A recent study [3], underlined that patient who underwent HSCT experienced clinically significant PTSD symptoms at 6 months after treatment, such as intrusion (re-experiencing the trauma), avoidance (with regard to cues of the traumatic event), and arousal (e.g., sleeping disturbances). The authors of the mentioned study identifies several baseline factors related with high risk of developing PTSD symptoms after HSCT. The study underscores the need for identifying patients at risk for PTSD symptoms to develop interventions and address the psychological impact of HSCT [3]. Lower baseline QoL, higher depression or anxiety before HSCT, and an increased anxiety during hospitalization, are all factors associated with higher PTSD symptoms at 6 months after HSCT [3]. It is well established that psychological distress is one of the most critical issues affecting survivorship care for patients with hematologic malignancies after HSCT [14]. Furthermore, the long-term psychological complications, with potential development of PTSD, can be related with non-adherence to medical treatments. In fact, the avoidance factor in PTSD patients implies that they avoid remembering the traumatic event with the elements associated with it, often including medications [10]. QoL can decrease in the first 30 to 100 days after the procedure, and it can improve by 1 to 2 years [15,16,17], but this longitudinal evidence is heterogeneous, with other investigations pointing out how the rates of PTSD can be completely independent of time and proposing PTSD as a chronic condition in some cases [18,19]. Although, as mentioned, transplantation techniques and supportive care have improved, diagnosis of PTSD symptoms has remained quite constant during the last two decades. Components related to the development of PTSD symptoms must be evaluated carefully with specific and tailored research design.

### 1.3. Graft-versus-Host Disease

As previously mentioned, an important factor associated with PTSD symptomatology after HSCT is the history of acute graft-versus-host disease (GvHD), a known major complication that occurs when donor bone marrow or stem cells attack the recipient, with obvious substantial influence on QoL in long-term survivors [20,21,22]. Ten years after undergoing HSCT, most patients report good to excellent QoL, compared to patients with chronic GvHD [23], conditioning regimen, gender, younger age, receiving less social support, and pre-transplant psychological distress were identified as factors able to predict lower overall health-related QoL [20]. 

### 1.4. Fatigue

Among the various psychological symptoms that may occur, with an impact on the perceived stress and trauma of this medical procedure, fatigue is of particular interest [18,24,25]. Indeed, although most long-term survivors after allo-HSCT recover adequately from treatment, a significant number of patients experience fatigue, which considerably reduces individuals’ QoL. Fatigue, evaluated from 28% to 35% at 3 years post HSCT [26,27], impacts negatively on life, and patients are constrained to down-regulate daily activities. Multiple challenges to address fatigue exist, including its multidimensional presentation, multiple causes, patient–clinician communication barriers, and few highly effective, evidence-based interventions that can be readily implemented [28]. Prevalence and severity of fatigue immediately following HSCT has been described as intense, with some cancer survivors reporting problems years after the treatment [29]. Compared with the general population of cancer survivors, fatigue remains persistently elevated among long-term HSCT survivors [30]. Up to 35% of long-term HSCT survivors report persistent moderate to severe fatigue even though they are disease free [27,31]. Three years after transplant, patients report significantly higher levels of fatigue compared to the general population [26], with moderate to severe fatigue found in 42% of a cross-sectional cohort of survivors 13 years (on average) after transplantation [32].

### 1.5. Aim of the Study

The aim of this study was to explore rates and risk factors for PTSD symptomatology, QoL, and fatigue in post-HSCT patients with hematological malignancies. We investigated which of these disease- or treatment-related factors predicted the severity of PTSD symptoms, and then we examined the specific role of fatigue. We expected to find a high percentage of patients with PTSD symptoms, according to the literature. We also hypothesized that PTSD symptoms would have been correlated closely with lower QoL. Finally, we explored the role of fatigue in this design.

## 2. Materials and Methods

### 2.1. Participants

A consecutive and unselected sample of 123 patients of the Hematology Unit, Pescara Hospital of Pescara (Italy), was recruited. All participants, male and female, had a medically documented diagnosis of hematological diseases, according to the diagnostic criteria of the World Health Organization. Patients were hospitalized in the Bone Marrow Transplant Unit, Department of Oncology-Hematology (Hospital of Pescara, Italy) and were contacted by clinical psychologists and medical hematologists. All patients met the following inclusion criteria: (a) adult patients with an age ranging from 18 to 65 years old; (b) patients with allo-HSCT older than one year. Conversely, patients younger than 18 years old, older than 65, with allo-HSCT more recent than one year, with severe addictions or neurological or psychiatric disorders, were excluded from the study.

### 2.2. Ethics 

The study was approved by the Ethical Committee with the name “Quality of life measurement in bone marrow transplantation: observational study on long-survivors patients” (code: QoL-BMT; 10/08/15; General Hospital Pescara, Italy). All recruited participants were informed about the scientific purpose of the study and gave their informed consent. The entire evaluation has been conducted as a regular clinical practice assessment of patients. All participants were assessed with both a gold standard interview and a psycho-diagnostic investigation [33], by clinical psychologists and physicians. Participants were volunteers; they filled out the questionnaires, they carried out the clinical interviews in a private setting and data were analyzed anonymously.

### 2.3. Study Measures 

Demographic data, including age, age at BMT, and gender, were collected in follow-up visits. The clinical outcomes, as well as years of therapy (BMT), diagnosis, and GvHD, were obtained from the patients’ medical records.

### 2.4. Impact of Event Scale-Revised (IES-R) 

The psychological distress was evaluated with the Impact of Event Scale-Revised (IES-R) [34], a 22-item self-report that measures the subjective distress caused by traumatic events. It consists of three subscales: Intrusion, Avoidance and Hyperarousal, associated with post-traumatic stress disorder (PTSD) symptoms. Participants were asked to rate the level of distress for each item during the previous seven days. The total IES-R score was graded from normal (0–23), mild (24–32), moderate (33–36), and severe psychological impact (>37). A cut-off score of 24 was used to define PTSD symptoms of clinical discomfort [34,35,36]. This 22-item scale has been used extensively in the literature, as 5 items were added to the original Horowitz (IES) scale to meet the American Psychiatric Association’s criteria for PTSD, although in this case we are careful, considering only symptoms of PTSD, not the clinical diagnosis of PTSD.

### 2.5. Functional Assessment of Chronic Illness Therapy-Fatigue (FACIT-F)

The Functional Assessment of Chronic Illness Therapy-Fatigue (FACIT-F) is a 13-item self-reported scale, where participants are asked to answer on a scale of one (not at all) to five (very much). It shows two items reversely scored, and overall scores of the FACIT-F scale range from 0 to 52, with higher scores signifying less fatigue [37].

### 2.6. Functional Assessment of Cancer Therapy-Bone Marrow Transplant (FACT-BMT)

Functional Assessment of Cancer Therapy-Bone Marrow Transplant scale (FACT-BMT) is a validated instrument for QoL measurement in bone marrow transplantation. It is a 50-item patient-reported questionnaire, which includes a 23-item BMT subscale and proposes a five points Likert-type scale ranging from 0 to 4. FACT-BMT measures 4 domains of well-being: physical (PWB), functional (FWB), social (SWB), emotional (EWB), and additional concerns (AC) specific to HCT. 

### 2.7. Statistical Analysis

Statistical analysis was conducted using STATA 14 [38]. A 3-step strategy was used for data analysis. First, socio-demographic and clinical variables between follow-up patients (with HSCT from 1 to 5 years) and long survivor patients (with HSCT from 6 to 10 years) were compared using Student’s *t*-test or Chi-Squared (χ^2^). The standardized mean difference was used as a measure of effect size. A standardized effect size (Cohen’s d) of 0.20–0.50 is considered small, 0.50–0.80 moderate, and >0.80 is considered large [39].

Second, binary logistic regression analysis was conducted to identify major determinants that best predict PTSD symptoms. PTSD symptomatology was measured as a dependent variable (dummy coded: 0 = without PTSD; 1 = with PTSD) and the independent variables were: age, age at BMT, gender, GvHD, years of BMT, QoL and fatigue. Three regression models were processed, and regression coefficients, the related confidence intervals, odds ratio, and *p*-values were estimated. In the first model, the three socio-demographic variables (age, age at BMT, and gender) describe the characteristics of patients. In the second model, clinical factors (GvHD and years of BMT) explaining the outcome, were integrated. Finally, in the third model, psychological variables were added (QoL and fatigue subscales). We considered how much each factor could add to the explained variance of the final model of PTSD symptoms.

Third, the Structural Equation Model (SEM) was used to assess the effect of latent constructs of QoL on the severity of PTSD symptomatology through the mediating role of fatigue. Our model included one exogenous latent trait (QoL), one endogenous latent factor for the PTSD items, treated as measurements loading (with the items as measurement loading), and one continuous mediator variables (fatigue). SEM is a set of statistical techniques used to measure and analyze the relationships of observed and latent variables. It examines linear causal relationships among variables, while simultaneously accounting for measurement error. SEM can be viewed as a combination of factor analysis and regression or path analysis. Latent factors represent the related theoretical constructs that can be considered latent traits or “true” variables underlying the measured items. The theoretical constructs in this study were QoL and fatigue, with their related and PTSD scores as latent traits. 

The measurement model can be of interest, but the focus of the investigation is usually set on the relationships among factors or between factors and observed variables (the structural part of the model) [40,41]. To evaluate the fit of the data to the model a maximum likelihood estimation method and the following multiple criteria was used: Chi-Squared (χ^2^) (*p* value > 0.05), Standardized Root Mean Square Residual (SRMR) value less than 0.08 is generally considered a good fit [42], Comparative Fit Index (CFI) near 0.90 or greater and Tucker–Lewis Index (TLI) near 0.90 or greater, is considered a good fit [43]. Hypotheses regarding the structural relationships among the constructs in the final model were evaluated using the magnitude of path coefficients (standardized coefficient) and their significance [44].

## 3. Results

### 3.1. Characteristics of the Sample

Of the 124 recruited patients, 123 (99.19%) were enrolled and there were no missing data. Most included patients were male (52.03%) and with a median age of 49.98 years and a median age at BMT of 42.01. Most of the participants had a diagnosis of Acute Myeloid Leukemia (AML) (40.65%) and 39.84% had GvHD. The mean of years since the BMT was 8.78. PTSD symptomatology was present in 58.54% of the sample.

### 3.2. Between-Group Comparisons

#### PTSD

Compared with the non-PTSD group, patients with PTSD symptomatology presented with GvHD (d = 0.46). No socio-demographic differences were found between the two groups. QoL dimensions such as physical, functional, social, emotional domain and further problems showed significant differences between groups, with effect sizes in the small–moderate range. Patients with PTSD symptomatology reported lower QoL (d = 0.61), and fatigue (d = 0.63) scores compared with patients without PTSD symptomatology (see Table 1).

### 3.3. Predicting PTSD Symptomatology from Quality of Life and Fatigue

Table 2 shows three regression models with PTSD score as a binary outcome criterion. In the first model, socio-demographic characteristics (age, age at BMT and gender) explained 4% of PTSD. Adding GvHD and years of disease produced a very small and not significant added predictor of PTSD of 5% (Model 2) with only GvHD presence showing the greater OR of 2.25 (95% CI [1.14, 6.43]). When the social dimension (OR = 1.10, 95% CI [1.02, 1.18]), and fatigue (OR = 1.20, 95% CI [1.11, 1.29]) were added in Model 3, they significantly explained an added 11% PTSD variance.

### 3.4. Structural Equation Model

**Figure 1:**
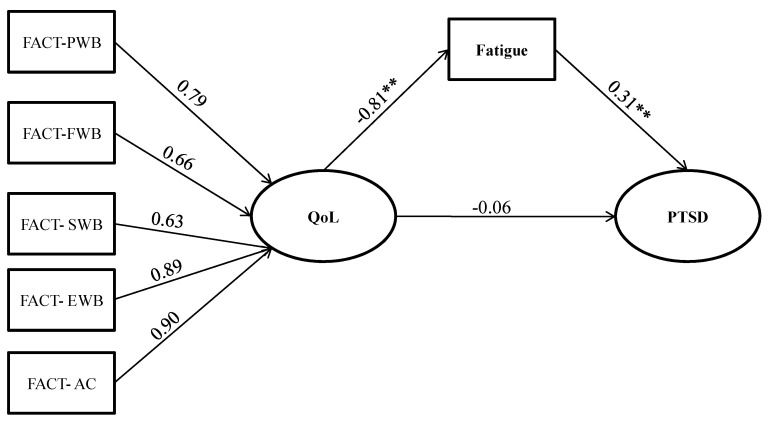
The mediation of PTSD symptoms. Note: Fact PWB: domains of well-being: physical; FWB: domains of well-being functional; SWB: domains of well-being social; EWB: domains of well-being emotional; AC: additional concerns; PTSD: Post Traumatic Stress Disorder; QoL: Quality of life; Path values are the path coefficients (standard errors). ** *p* < 0.01.

In support of the binary logistic regression analysis, SEM analyses were performed to test the direct and indirect effects in a mediation model of latent dimension of QoL on PTSD mediated by fatigue symptoms. The structural components of the model included one exogenous latent trait (QoL), one endogenous latent factor for PTSD, and one continuous mediator variable. Figure 1 shows the path analysis and parameter estimates. All the observed variables were loaded on their corresponding latent constructs, supporting the validity of the construct of each latent construct, and standardized residuals were normally distributed. The parameter model estimates indicated that QoL exerted a significant direct negative effect on Fatigue (β = −0.81), whereas there were no significant direct effects of PTSD (β = −0.06). Fatigue has a positive significant effect on PTSD (β = 0.31). The higher the QoL the lower the fatigue, in the same way a subject with high levels of fatigue shows a greater presence of PTSD.

The significant indirect effects are shown in Table 3. SEM showed a negative effect of QoL on PTSD symptomatology through the mediation of fatigue symptoms (β indirect = −0.33, *p* < 0.001). QoL significantly influenced PTSD symptoms exclusively through the mediation role of fatigue. 

The values of multiple fit indices indicated that the proposed model provided good fit data, (χ^2^ = 45.478, df = 13, *p* = < 0.001, TLI = 0.90, CFI = 0.93, CD = 0.92, and SRMR = 0.03).

In sum, the SEM analysis showed that the QoL dimensions affected PTSD along different pathways. QoL was found to be a major factor influencing PTSD not directly, but through the mediation role of fatigue.

## 4. Discussion

“As Supelana and colleagues proposed [10], transplant can be seen as a “scheduled trauma”, therefore presenting a unique feature among other traumatic experiences since they are “not scheduled” by definition”. HSCT has even more remarkable features, being the only not-solid transplant, less explored in its physical, psychological, and specifically traumatic characteristics. As previously mentioned, despite the enormous increase in the number of transplants and the improvement of their medical quality, general psychophysical health conditions of patients have not much improved. These patients are underrepresented in studies of traumatic experiences. 

First, we hypothesized that an association exists between HSCT and PTSD symptoms in these patients. Then, we explored factors able to predict PTSD symptoms severity, assuming that higher PTSD symptomatology was associated with lower QoL. Finally, we examined the role of fatigue in this design, assuming its importance. 

As previously mentioned, more than half of patients (57%), with specific criteria, can develop symptoms of PTSD [5], a percentage confirmed by our research with a value of 58.54%, confirming our first hypothesis. We have also shown that individuals with PTSD symptomatology reported significantly lower QoL total scores, confirming our second hypothesis. Moreover, we tried to answer our third explorative question about the role of fatigue; we found that it has a significant effect on PTSD symptoms, showing a negative correlation with QoL. In other words, individuals with high levels of fatigue have a lower QoL and greater presence of PTSD symptoms. In fact, our SEM has shown the mediation power of fatigue on QoL, influencing the PTSD outcome.

Research in QoL in these clinical populations is important for different reasons. First, research in this field might help patients and medical staff comparing the objective outcome of transplantation therapies against subjective QoL expectations and assessment [45]. This could represent a step forward in the meaningful involvement of patients in clinical decision making. Second, research in QoL aims to develop patient-orientated rehabilitation, or recovery, programs [17]. Finally, since this traumatic experience is programmed in advance, research can explore specific patients’ characteristics to lower the traumatic expression of the procedure, with more personalized intervention, taking into account the individual characteristics of patients [42].

Psychiatry, psychology, and psychoanalysis started taking an interest in these phenomena (transplants) decades ago, studying the traumatic reactions of transplant patients, discovering that the frequency of serious psychiatric complications has been higher than in any other group of surgical patients [46]. PTSD is in fact composed of an intertwining of psychophysical components, where fatigue can play an important role. Among all psychiatric disorders, PTSD shares the strongest relationship with somatization and, particularly, medically unexplained pain [47].

The effects of stress on the hypothalamic pituitary adrenal axis and the autonomic nervous system have been explored and explained by the “allostatic load” as an attempt to regulate psychophysical stress. The physiological dysregulation that underpins allostasis represents a final common pathway to disease that can be manifest in various ways [48], including fatigue [49], as we confirmed in our study. Even though we did not explore dysfunction of the hypothalamic pituitary adrenal axis, this factor has a known key role in the onset of chronic widespread musculoskeletal pain also in a general population sample [48].Cancer-related fatigue is a common and persistent concern for cancer survivors [50], including HSCT patients [27], and this symptom has also shown an association with cognitive concerns for HSCT patients [51,52]. Furthermore, fatigue and physical complaints (among other symptoms) are common long-term reactions even in different traumatic experiences, such as physical and sexual abuse [50], confirming the association between psychological (traumatic) experience and physical (fatigue) complaints.

Neuroimaging studies on patients with chronic fatigue have used functional connectivity, showing lower functional connectivity at the prefrontal cortex, both during rest and cognitive task [53,54]. Other evidence also shows an inefficient increase in resting-state functional connectivity linked to the psychological factors observed in the syndrome [55].

According to Freud (1938), physical symptoms can be a way for the body to communicate, a request of care of the patients who need to be heard [56]. In fact, recent studies have shown that psychotherapy can improve symptoms such as fatigue, insomnia, and depression in cancer patients [57,58,59]. This view is consistent with current approach in psychology, cognitive, and psychodynamic neuroscience [60,61,62] and with the classical view of the embodied mind, or embodied cognition [63], in which the mind makes essential demands on bodies, where the brain is not the exclusive cognitive resource we possess to solve problems [64].

QoL studies, specifically designed to identify factors associated with poor QoL, are important in recognizing areas that can be improved in the provision of information, support and clinical care to patients undergoing allogeneic HSCT, including patient-orientated psychophysical rehabilitation programs, especially in patients more prone to develop PTSD.

Our study has limitations: firstly, our sample size is relatively small, and it needs a larger sample to confirm our findings. Additionally, our cross-sectional design limits the ability to determine role and direction of the association between potential risk factors and PTSD symptoms, making a longitudinal approach necessary. Moreover, the length the length of hospital stay should be investigated as another risk factor in the development of PTSD symptoms [65]. In our study, we were unable to recollect this information for all our patients. Finally, the questionnaires used to ascertain PTSD symptoms displayed sub-optimal discriminatory characteristics compared to structured and semi-structured diagnostic interviews.

However, our research also has strengths: to the best of the authors’ knowledge, no studies have investigated the pathways through which QoL can influence post-traumatic stress disorder in hematological patients, especially in a not-solid transplant procedure, where the number of these patients is not remarkable, diminishing the impact of our first limitation. Our current study offers valuable information about the prevalence of PTSD symptoms, in a specific sample, underlining the role of fatigue in this clinical population.

## Figures and Tables

**Table 1:** Socio-demographic and clinical characteristics between groups: with and without PTSD symptoms.

		Total Sample (N = 123)	PTSD N = 72	Non-PTSD N = 51	*t/χ* ^2^	*p*	*d*
Age		49.98 (14.67)	49.66 (14.01)	50.43 (15.69)	0.28	0.77	0.05
Age at BMT		42.01 (15.42)	41.84 (14.49)	42.25 (16.79)	0.14	0.88	0.02
Gender	Male	64 (52.03%)	39 (54.17%)	25 (49.02%)	0.57	0.55	0.10
	Female	59 (47.97%)	33 (45.83%)	26 (50.98%)			
GvHD	No	74 (60.16%)	37 (51.39%)	37 (72.55%)	2.39	<0.01	0.46
	Yes	49 (39.84%)	35 (48.61%)	14 (27.45%)			
Diagnosis	ALL	18 (14.63%)	12 (16.67%)	6 (11.76%)			
	AML	50 (40.65%)	27 (37.5%)	23 (45.10%)			
	CML	20 (16.26%)	12 (16.6%)	8 (15.69%)			
	MDS	14 (11.38%)	10 (13.8%)	4 (7.84%)			
	MM	3 (2.44%)	3 (4.17%)	0			
	NHL	7 (5.69%)	1 (1.39%)	6 (11.76%)			
	THAL	11 (8.94%)	7 (9.72%)	4 (7.84%)			
Years from BMT		8.78 (7.80)	8.65 (7.09)	8.98 (8.77)	0.22	0.81	0.004
Fact PWB		23.13 (5.50)	22.08 (6.07)	24.60 (4.22)	2.56	<0.01	0.46
FactSWB		20.81 (6.05)	19.81 (6.46)	22.23 (5.14)	2.21	<0.01	0.40
FactEWB		18.77 (4.01)	17.86 (4.04)	20.05 (3.64)	3.09	<0.001	0.56
FactFWB		19.17 (6.67)	17.75 (7.20)	21.19 (5.29)	2.90	<0.001	0.53
FactAC		31.06 (6.16)	28.98 (6.88)	32.29 (5.18)	2.89	<0.001	0.52
FACT total score		111.9 (24.4)	106.01 (27.1)	120.3 (17.04)	3.33	<0.001	0.61
Fatigue		40.65 (11.3)	37.80 (12.5)	44.68 (7.80)	3.46	<0.001	0.63

Note: BMT: Bone Marrow Transplant; GvHD: graft-versus-host disease, Fact PWB: domains of well-being: physical; FWB: domains of well-being functional; SWB: domains of well-being social; EWB: domains of well-being emotional; AC: additional concerns.

**Table 2:** Variables significantly predicting severity of PTSD symptoms: results from binary logistic regression models.

	β	SE	OR (95% C.I.)	*p*-Value	R^2^
Model 1					0.04
Age	0.08	0.10	0.97 (0.90–.1.05)	0.55	
Age at BMT	0.11	0.09	1.00 (0.93–1.07)	0.89	
Gender	0.78	0.25	0.62 (0.60–1.70)	0.36	
Model 2					0.09
+GvHD	0.28	0.59	2.25 (1.14–6.43)	<0.01	
+Years of BMT	0.10	0.08	0.91 (0.76–1.08)	0.28	
Model 3					
+Fact-PWB	0.07	0.09	0.92 (0.70–0.98)	0.43	0.20
+Fact-SWB	0.19	0.07	1.00 (0.98–1.05)	<0.05	
+Fact-EWB	0.13	0.08	0.87 (0.71–1.06)	0.19	
+Fact-FWB	0.03	0.09	1.03 (0.86–1.23)	0.71	
+Fact-AC	0.05	0.09	1.06 (0.88–1.26)	0.45	
+Fatigue	0.16	0.04	1.00 (0.92–1.09)	<0.01	

Note: BMT: Bone Marrow Transplant; GvHD: graft-versus-host disease, Fact PWB: domains of well-being: physical; FWB: domains of well-being functional; SWB: domains of well-being social; EWB: domains of well-being emotional; AC: additional concerns.

**Table 3:** Indirect effects on PTSD (N = 123).

	β	SE	*p*	z
Fatigue				
QoL (FACT total score)	−0.33	0.07	<0.05	1.94

Note: QoL: Quality of life.

## Data Availability

Data is unavailable due to privacy or ethical restrictions.
